# People’s experiences of their involvement in nursing care: a systematic review protocol

**DOI:** 10.3399/BJGPO.2024.0048

**Published:** 2024-10-02

**Authors:** Diana Santos, Eduardo Santos, António Fernando Amaral

**Affiliations:** 1 Univ Coimbra, Health Sciences Research Unit: Nursing (UICISA: E), Nursing School of Coimbra (ESEnfC), Hospitais da Universidade de Coimbra, Unidade Local de Saúde de Coimbra, EPE, Coimbra, Portugal; 2 Health Sciences Research Unit: Nursing (UICISA: E), Nursing School of Coimbra (ESEnfC), School of Health, Polytechnic University of Viseu, Portugal Centre for Evidence-Based Practice: A JBI Centre of Excellence, Coimbra, Portugal; 3 Health Sciences Research Unit: Nursing (UICISA: E), Nursing School of Coimbra (ESEnfC), Coimbra, Portugal

**Keywords:** Hospitals, Nursing care, Patient participation

## Abstract

**Background:**

People's involvement and participation in their own care are the essential basis of nursing care. This phenomenon can be characterised as an approach based on the integration of the person’s values, beliefs, and preferences during nursing care. This process contributes to improve quality of care, improve satisfaction levels, and result in a better experience for people receiving care. To promote the person’s participation in nursing care, it is necessary to better understand their experiences about this topic.

**Aim:**

To synthesise the available evidence on people’s experiences of their involvement and participation in nursing care in a hospital setting.

**Design & setting:**

A systematic review that will be conducted according to the JBI methodology for systematic reviews of qualitative evidence.

**Method:**

The study selection, critical appraisal, and data extraction will be conducted by two independent reviewers. This review will consider studies with a qualitative approach, published and unpublished, in Portuguese, English, or Spanish, with no temporal limit, which include adults, aged 18 years or older, who have experienced an admission to a hospital, that explored people’s experiences of their involvement and participation in nursing care in hospital ward settings. Findings will be presented using a meta-aggregation approach and narrative format, and the final synthesised findings will be graded according to the ConQual approach.

**Conclusion:**

It is expected that this qualitative synthesis will inform people, health professionals, and policymakers, allowing them to develop recommendations to promote the person’s participation in nursing care.

## How this fits in

It is known that the involvement and participation of people in nursing care improves the quality of care, levels of satisfaction, and the experiences of the people who receive this care. Understanding people’s experiences on this topic will help to promote people’s participation in nursing care.

## Introduction

Global health organisations advocate people’s involvement and participation in health care and health policies as an international priority.^1–3^ The involvement and participation of the person in health care are integrated into the paradigm of person-centred care.^4^ In 2015, the World Health Organization re-emphasised the need for person-centred care,^5^ which is characterised by a philosophy of care that integrates the perspectives, culture, values, and preferences of individuals, families, and communities.^4,5^

The active position of the person in their health–disease process has been increasingly highlighted.^1,5^ Specifically, in nursing practice, the people’s involvement and participation are essential.^6,7^ This process can be characterised as an approach based on the integration of the person’s values, beliefs, and preferences during nursing care.^6–8^ It is described as a dynamic process between nurses and people who receive nursing care, which requires the establishment of a relationship, with sharing of power and knowledge, and their mutual involvement.^6–8^ From this point of view, it is argued that a person's participation in their care can only happen when there is mutual trust, respect, and commitment between nurses and people.^6–9^

Several studies describe that this approach promotes individualised care, based on a partnership between nurses and people who receive nursing care.^6–11^ Thus, the person is involved and taken into account in decision-making regarding the provision of nursing care.^6–8^

Authors have reported that the involvement and participation of the person in nursing care promotes better health outcomes for the person, improves satisfaction levels, and improves the person’s experience of hospitalisation.^10^ Other authors have reported that it contributes to safe care and the prevention of adverse events.^12–14^ Above all, this process contributes to improving the quality of care.^13^

However, people’s involvement and participation in nursing care are considered a challenge and some barriers are perceived by people who experienced it during their stay in the hospital setting.^14,15^ For example, in one study, some informants considered it a self-related barrier that they preferred not to participate or get involved (to play a passive role) and listed the following as reasons: the overload of the disease and their experience; low knowledge of health care; and low self-esteem.^15^ Also, the lack of empathy and compassion between people and nurses, the centralised power of nurses, the organisational and human resources characteristics (for example, different nurses on each shift),^15^ and gaps in teamwork were identified as barriers to this process.^14^

To overcome these barriers, the authors underlined that nurses have a crucial role in promoting person participation.^11^ This is in alignment with Tobiano *et al*,^16^ whose study about the perception of these professionals on this topic, they reported that people should be considered partners, and reinforced the importance of this partnership for safety in care. However, the people’s characteristics, namely their attitude and willingness to be involved and participate in nursing care, were considered a difficulty in this process by the nurses.^16^ In the same line of thought, authors pointed out that the involvement and participation of the person in health care was difficult to understand because some people may prefer to be more passive (in other words, to give the other person the ability to decide), which adds a complexity in the understanding and implementation of their involvement and participation.^17^ Therefore, to promote people’s participation and involvement in nursing care, a better understanding of their experiences on this process is necessary. There are studies that have aimed to explore people’s experiences of their involvement and participation in nursing care in a hospital setting because of the importance described above.^9,15,17–19^

Thus, once evidence-informed health care considers the experience of the person being cared for, in addition to the best available evidence, the care context, and the professional’s critical judgement.^20^ It is therefore necessary to synthesise the evidence about people’s experiences of their involvement and participation in nursing care in a hospital setting.

A preliminary search of PROSPERO, MEDLINE (PubMed), CINAHL (EBSCO), and JBI Evidence Synthesis was conducted on 9 February 2024. In this search, one review on this topic was identified that aimed to investigate people’s and nurses’ perceptions and behaviours towards person participation in nursing care in the context of hospital medical wards, however, this review will restrict the context to medical wards (excluding other wards, such as surgery) and has some methodological limitations, for example, the authors described the design as an integrative review, which lacked a sustained methodological framework, and the database searches were conducted with only index terms.^21^

The objective of this review is to synthesise the available evidence on people’s experiences of their involvement and participation in nursing care in a hospital setting.

## Method

A systematic review will be conducted according to the JBI methodology for systematic reviews of qualitative evidence.^22^ The Preferred Reporting Items for Systematic Reviews and Meta-Analyses Protocols (PRISMA-P) was followed to develop this protocol.^23^ This review is registered in PROSPERO (CRD42024506501).

### Eligibility criteria

The eligibility criteria were developed according to the PICo framework (Participants, Phenomena of interest, and Context).^22^ This review will consider studies that include adults, aged 18 years or older, who have experienced an admission to hospital. Studies with people who needed maternity care, ambulatory care, palliative care, psychiatric care, emergency care, and intensive care will be excluded. Regarding phenomena of interest, this review will consider studies that explore people’s experiences of their involvement and participation in nursing care. People’s involvement and participation in nursing care is characterised as making people partners in care (from the planning to the implementation and evaluation of nursing care).^6,7^ This process requires information sharing, power sharing, and an established relationship between nurses and people who receive nursing care.^6,7^ With regards to context, studies that have been developed in hospital wards settings will be considered. Studies conducted in ambulatory care, maternity department, emergency department, intensive care, operating room, palliative care, and psychiatric settings will be excluded because of the specificities of these contexts.

This analysis will consider studies based on the interpretative paradigm, such as descriptive-exploratory studies with a qualitative approach, phenomenology, grounded theory, ethnography, action research, or feminist research.

### Search strategy

A comprehensive search (a balance between sensitivity and specificity) will be conducted to identify published and unpublished studies for this systematic review. Initially, a search was conducted in MEDLINE (PubMed) and CINAHL Complete (EBSCOhost) with the keywords: “nursing care”, “patient participation”, and “patient involvement” (and other combinations with only two or three expressions). Through this initial search, some relevant studies were identified. The words contained in the titles and abstracts of these articles, as well as the index terms, were used to develop a concept map. With the support of this map, a comprehensive search strategy was constructed. A complete search strategy in the MEDLINE (PubMed) database can be found in Supplementary Table S1. The reference list of all the studies selected for critical appraisal will be analysed for further studies.

To identify published studies, the following databases (and their respective search platforms) will be considered: MEDLINE (PubMed), CINAHL Complete (EBSCOhost), MedicLatina (EBSCOhost), Psychology and Behavioural Sciences Collection (EBSCOhost), and SciELO. Sources of unpublished studies and grey literature to be searched include MedNar, Repositório Científico de Acesso Aberto de Portugal (RCAAP–Open Access Scientific Repositories of Portugal), ProQuest Dissertations and Theses Global, and the first 10 pages of Google Scholar (because of the large number of results).

Studies published in Portuguese, English, or Spanish will be included. Studies with full text only in other languages will be excluded, however, they will be reported in the final review. Thus, for search strategy, studies published in any language will be considered, without any publication date limit.

### Study selection

After searching for published and unpublished studies in all the above-mentioned resources, all the records will be uploaded to Mendeley (version 1.19.8). In this software, duplicates will be removed, and after this process, these records will be imported into Covidence (online systematic review software by Veritas Health Innovation, Melbourne, Australia). Before the study selection process begins, the team of reviewers will organise a meeting to assess and increase understanding of the inclusion criteria and to reflect on possible doubts.

Regarding the study selection, firstly, titles and abstracts will be analysed by two independent reviewers (DS and ES). After that, the full texts of potentially relevant records will be recovered and will be assessed by two independent reviewers (DS and ES). This assessment of titles, abstracts, and full texts will be based on the defined inclusion criteria. In the process of study selection, any disagreements that arise between the reviewers at each stage of the study selection process will be resolved through discussion or with a third reviewer (AFA; we will conduct team reflection meetings whenever necessary). Also, when only the title and/or abstract is found, the authors will be contacted to request the full text (if it is available) or data, in the event of ongoing research. The reasons for exclusion will be reported in the systematic review report.

The results will be reported in full in the final systematic review and presented in a Preferred Reporting Items for Systematic Reviews and Meta-analyses (PRISMA) flow diagram.^24^

### Assessment of methodological quality

Eligible studies (included after full text analysis) will be critically appraised by two independent reviewers (DS and ES) for methodological quality. For this assessment, the JBI critical appraisal checklist for qualitative research will be used.^25^ Before this phase, a meeting will be conducted with all the reviewers to assess the comprehension of the assessment instrument (discussion of possible doubts and development of an example). In the process of assessing methodological quality, any disagreements that arise between the reviewers will be resolved through discussion or with a third reviewer (AFA. We will conduct team reflection meetings whenever necessary). When necessary, due to missing data or the need for additional clarification on methodological aspects, the authors of the studies will be contacted. Regardless of the methodological quality of each study, all will be included. The results of the assessment of the methodological quality of each study will be presented in a table with narrative support.

### Data extraction

Data will be extracted from studies included in the review by two independent reviewers (DS and ES) using the standardised JBI data extraction tool.^22^ The data extracted will include specific details about study description: date of publication, authors, journal, methods, phenomena of interest, setting/context (description of specific context in the hospital), geographical/cultural, participants' characteristics (age, sex [biological], cause of hospitalisation), data analysis (type of qualitative analysis), conclusions, and others relevant information (reflexivity and research team, and other aspects related to the rigour of qualitative research). Findings and their illustrations (voice of the informants) will be extracted and assigned a level of credibility: 'unequivocal' (finding with illustration that is unquestionable), 'equivocal' (finding with illustration that can be contested), or 'unsupported' (finding not supported with illustration).^26^ In the process of data extraction, any disagreements that arise between the reviewers will be resolved through discussion, or with a third reviewer (AFA. We will conduct team reflection meetings whenever necessary). Before this phase, a meeting will be conducted with all the reviewers to assess the comprehension of the data extraction tool (discussion of possible doubts and development of an example). When necessary, due to missing data or the need for additional clarification, the authors of the studies will be contacted.

### Data synthesis

Qualitative research findings will, where possible, be pooled with the meta-aggregation approach.^22,25^ Only unequivocal and equivocal findings will be included in the aggregation, and findings not supported with illustration will not be considered, and when aggregation is not possible, the findings will only be presented in narrative format. The final synthesised findings will be graded according to the ConQual approach.^26^

## Discussion

This qualitative systematic review will provide a rigorous synthesis of people’s experiences of their involvement and participation in nursing care that will inform people who receive nursing care, academics, and health professionals. This review will provide a comprehensive understanding of this phenomenon and it is expected that recommendations will be developed to promote the person’s participation in nursing care and consequently all the associated benefits.

## Trial registration number

PROSPERO CRD42024506501

## Data

Data produced through this research will be available from the corresponding author on reasonable request.
